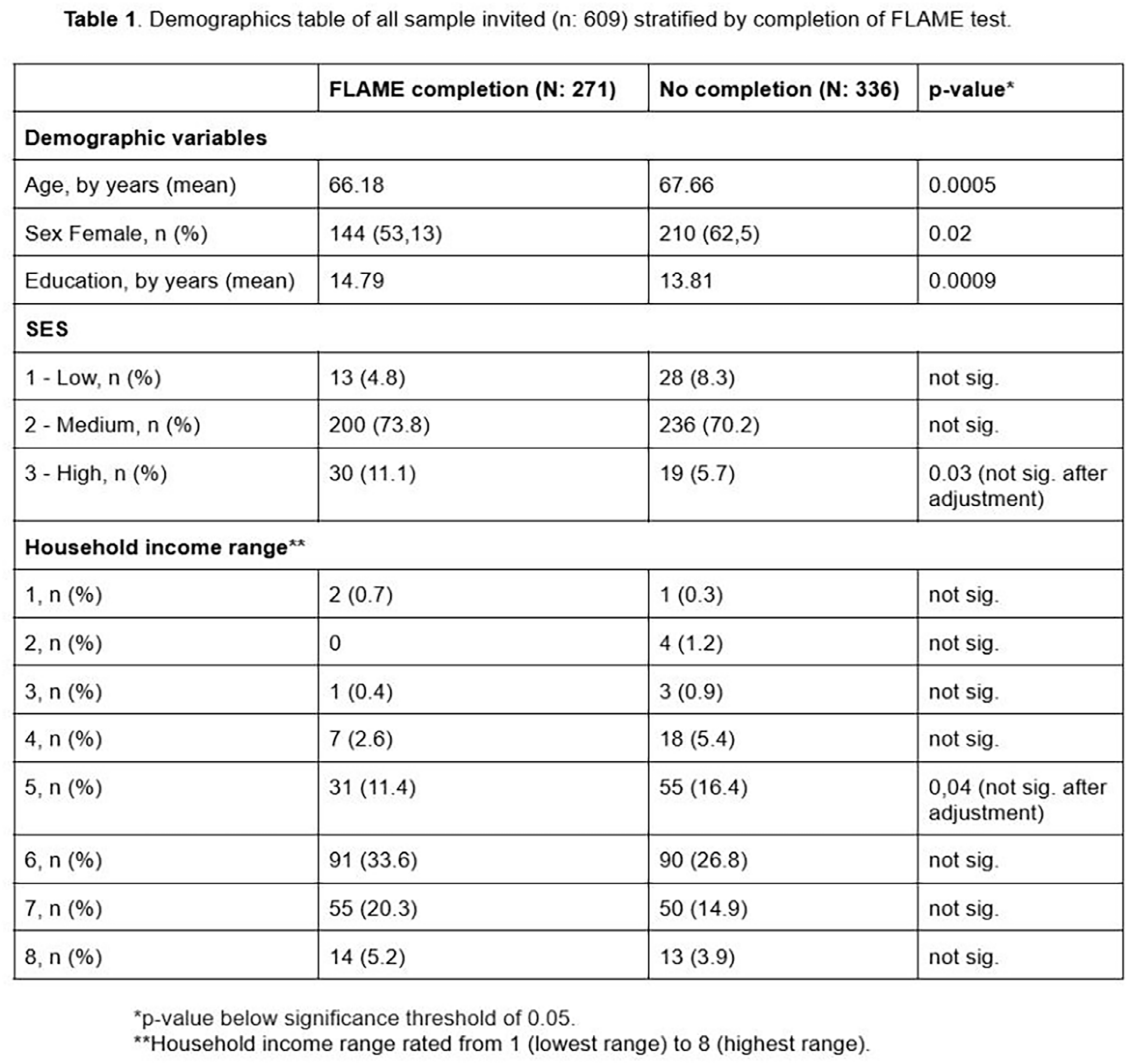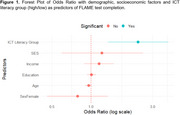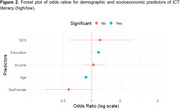# Predictors associated with the rate of completion of a remote cognitive assessment

**DOI:** 10.1002/alz70856_105560

**Published:** 2026-01-10

**Authors:** Clàudia Porta‐Mas, Gonzalo Sánchez‐Benavides, Anna Brugulat‐Serrat, Andreea Rădoi, Mireia Sánchez‐Guitérrez, Karine Fauria, Anna Soteras, Anne Corbett, Juan Domingo Gispert, Oriol Grau‐Rivera

**Affiliations:** ^1^ Barcelonaβeta Brain Research Center (BBRC), Pasqual Maragall Foundation, Barcelona, Spain; ^2^ Hospital del Mar Research Institute, Barcelona, Spain; ^3^ Centro de Investigación Biomédica en Red de Fragilidad y Envejecimiento Saludable (CIBERFES), Instituto de Salud Carlos III, Madrid, Spain; ^4^ University of Exeter, Exeter, United Kingdom; ^5^ Servei de Neurologia, Hospital del Mar, Barcelona, Spain

## Abstract

**Background:**

Remote cognitive assessments offer a scalable and efficient alternative to traditional paper‐and‐pencil tests for large‐scale research and clinical studies. However, factors such as digital literacy, demographics, and socioeconomic status may influence participant engagement. This study examined the influence of these factors on response rate (test completion) in the web‐based FLAME cognitive assessment among non‐demented participants in BBRC observational cohorts.

**Method:**

609 participants (mean[SD] age:67[5.3]; 354 women; mean[SD] education:14.2[3.6]) were invited by e‐mail to complete FLAME. Previously, they had been invited to complete an ad‐hoc questionnaire assessing digital literacy, which included seven self‐rated items on familiarity and comfort with ICTs use. Based on a median split, participants were classified as having high or low ICT literacy. Age, sex, years of education, household income, subjective socioeconomic status (SES) (low, medium, or high), and living area within Catalonia, (levels: highly populated, medium populated, and rural) were also collected. Univariate and multivariate logistic regression models were used to examine if digital literacy, demographic, and socioeconomic factors predicted test completion. Additionally, predictors of digital literacy were explored using demographic and socioeconomic variables.

**Result:**

The FLAME test completion rate was 44.49%. High ICT literacy emerged as the strongest predictor of FLAME test completion (OR: 2.31; *p* =  0.002), independent of age, sex, education, income, and SES. In a multivariate model assessing demographic and socioeconomic as predictors of ICT literacy, younger age (OR: 0.87; *p* =  <0.0001) and higher education (OR: 1.18; *p* =  <0.0001) were significant predictors of higher ICT literacy, while female sex (OR: 0.57; *p* =  0.051) showed a strong trend toward lower ICT literacy, and income and SES were not significant. Although living area did not significantly influence ICT literacy, participants from rural areas had a higher FLAME completion rate (72.4%) compared to those from medium‐density (50%) and high‐density (54%) populated areas.

**Conclusion:**

Higher digital literacy was significantly associated with higher response rates in remote cognitive assessments, emphasizing the importance of assessing it beforehand. Additionally, demographic and socioeconomic factors may influence digital literacy and should be considered in these evaluations.